# Analyse biochimique d’un pic insolite d’allure monoclonale en électrophorèse des protéines urinaires: à propos d’un cas

**DOI:** 10.11604/pamj.2022.41.53.25726

**Published:** 2022-01-19

**Authors:** Aboubacar Dit Tietie Bissan, Raoul Karfo, Amadou Diawara, Aboubacar Tekete, Oumar Tangara

**Affiliations:** 1 Centre d’Infectiologie Charles Mérieux, Bamako, Mali,; 2 Université des Sciences, des Techniques, des Technologies de Bamako, Faculté de Pharmacie, Bamako, Mali,; 3 Unité de Formation et de Recherche (UFR) Sciences de la Santé, Université Ouaga Joseph KI-ZERBO, Ouagadougou, Burkina Faso,; 4 Laboratoire d´Analyses Médicales Algi, Quinzambougou, Bamako, Mali

**Keywords:** Electrophorèse, protéines urinaires, protéinurie de surcharge, myoglobinurie, cas clinique, Electrophoresis, urine protein, overload proteinuria, myoglobinuria, case report

## Abstract

L´électrophorèse des protéines urinaires est souvent nécessaire au diagnostic et au suivi de certaines pathologies urologiques ou rénales, et des hémopathies lymphoïdes. Nous rapportons le cas d´un profil atypique d´électrophorèse des protéines urinaires sur capillaire et en gel d´agarose. Il s´agit d´un pic de nature inconnue d´allure monoclonale migrant au niveau des gammaglobulines. La littérature et les dosages effectués prouvent qu´il s´agissait de la myoglobine. En effet, la myoglobine de 17,5 kDa est librement filtré par le glomérule, et normalement réabsorbé au niveau tubulaire. En cas de dépassement de sa capacité de réabsorption, sa présence entraine une protéinurie de surcharge. Cette myoglobinurie nous a permis de mettre en évidence une rhabdomyolyse aigüe chez notre patient. Ainsi, l´analyse des pics inconnus comme dans ce cas, renseigne sur des symptomatologies, mais aussi des pathologies sous-jacentes, qui peuvent avoir un intérêt clinique.

## Abstract (en)

Urine protein electrophoresis is often required for diagnosis and monitoring of urological or renal diseases and lymphoid hemopathies. We here report an uncommon urine protein electrophoresis result. The test was performed using agarose gel electrophoresis and capillary electrophoresis. It was a monoclonal peak of unknown significance migrating with gammaglobulins. Scientific literature and the tests performed demonstrated that it was myoglobin. In fact, myoglobin (17 kDa) is freely filtered by the glomerulus and normally reabsorbed by the tubules. If tubule capacity for reabsorption is exceeded, its presence results in overcharging proteinuria. Myoglobinuria helped diagnose rhabdomyolysis in our patient. Thus, the analysis of unknown peaks, can provide information on symptoms but also underlying pathologies, which may be of clinical interest.

## Introduction

L´électrophorèse des protéines urinaires est en phase de devenir une analyse de routine en laboratoire d´analyses médicales. Elle nécessite cependant du matériel spécifique. Cette électrophorèse constitue un examen le plus souvent prescrit par un médecin spécialiste, néphrologue, interniste, ou autres lorsque celui-ci soupçonne soit une gammapathie monoclonale, soit une néphropathie glomérulaire ou une tubulopathie proximale [1]. Ainsi, les protéinuries sont associées à des pathologies multiples ayant des causes très diverses. Le rôle des renseignements cliniques dans l´ensemble de ces contextes est indispensable pour une bonne interprétation des résultats par le biologiste. Nous rapportons un cas de profil atypique d´électrophorèse capillaire des protéines urinaires de découverte fortuite.

## Patient et observation

**Information relative au patient:** il s´agit de T.H, homme âgé de 65 ans qui a consulté à cause de vertiges récidivants.

**Résultats cliniques:** l´examen clinique a mis en évidence des conjonctives moyennement colorées et un abdomen souple sans masse ni organomégalie.

**Chronologie:** les vertiges ont continué pendant un mois avant le retour du patient au service d´oncologie pour la suite de la prise en charge.

**Figure 1:**
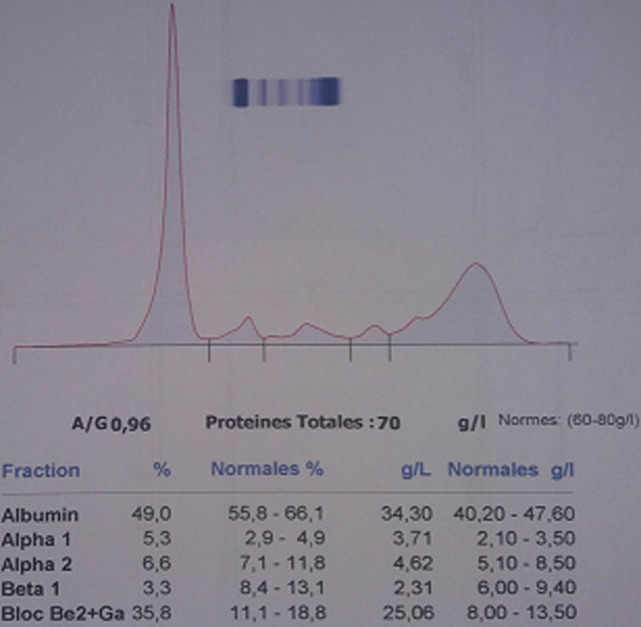
tracé d’électrophorèse capillaire des protéines sériques

**Figure 2:**
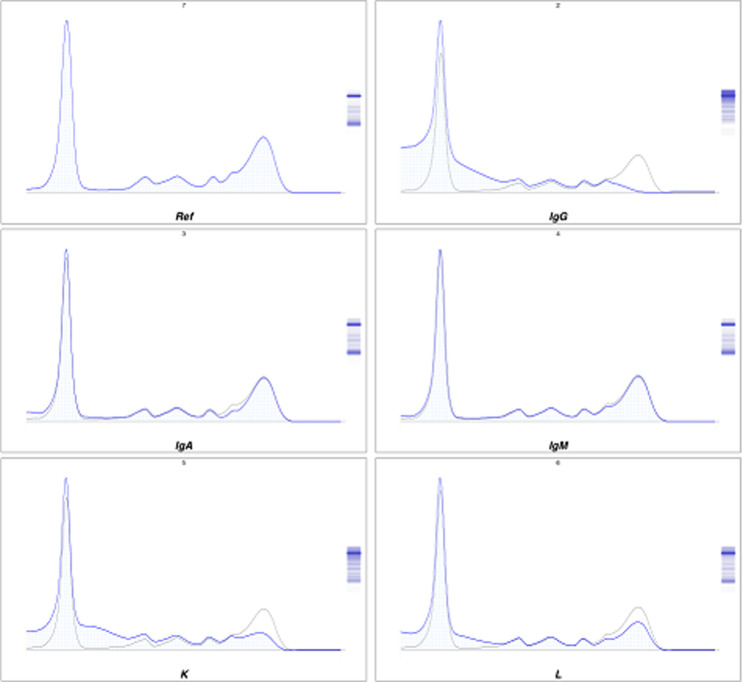
immunotyping du sérum

**Figure 3:**
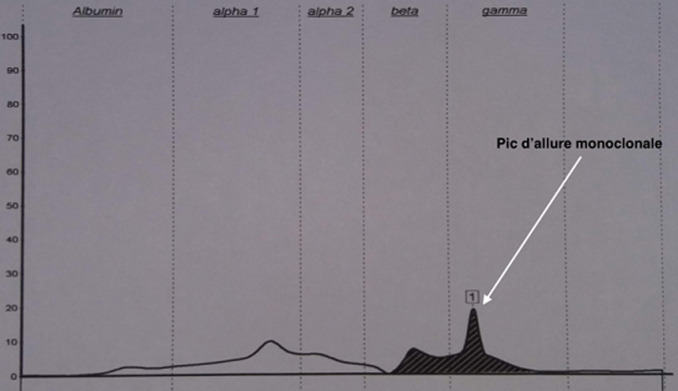
tracé d’électrophorèse capillaire des protéines urinaires

**Figure 4:**
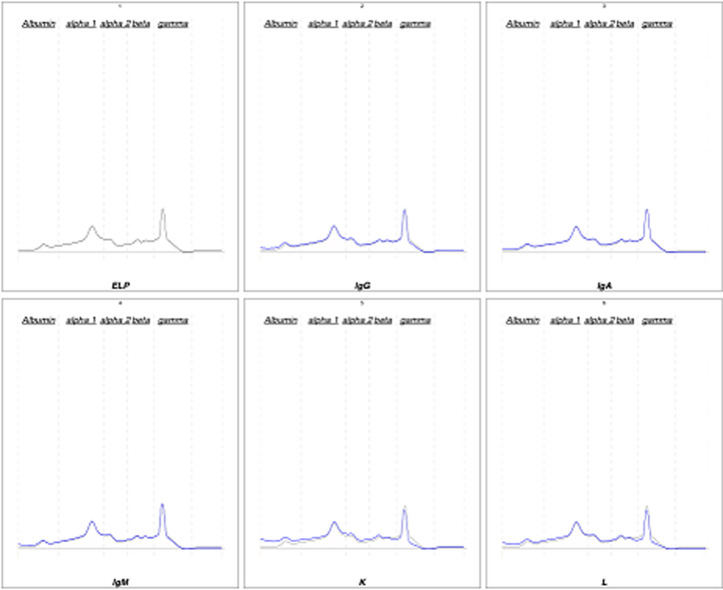
immunotyping urinaire

**Figure 5:**
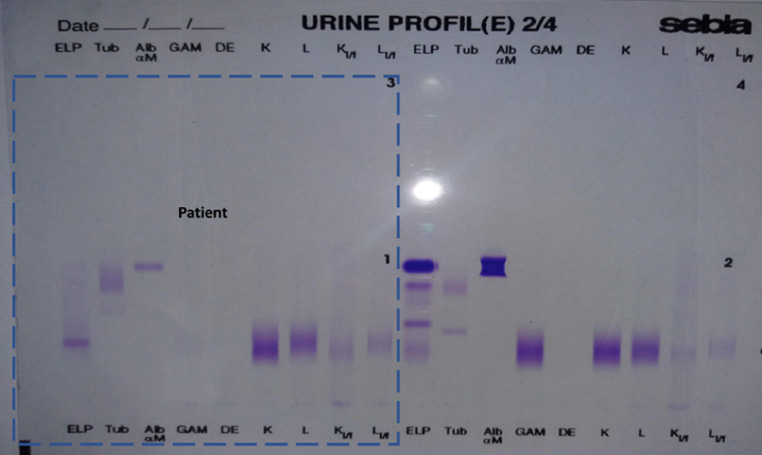
immunofixation urinaire

**Figure 6:**
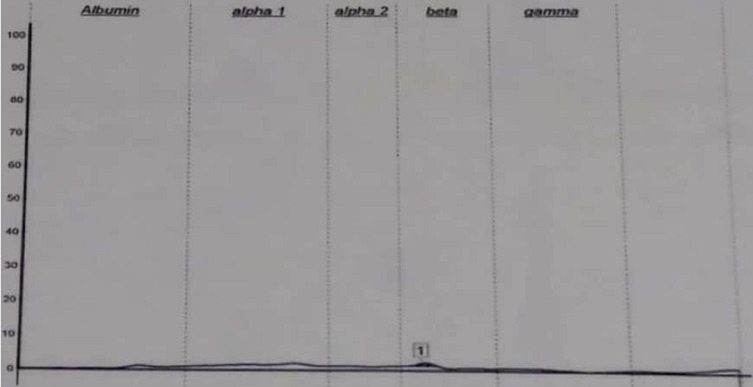
tracé d’électrophorèse capillaire des protéines urinaires après un mois

**Démarche diagnostic:** le patient a été adressé au laboratoire pour la réalisation d´une électrophorèse des protéines sériques et urinaires avec une recherche des protéines de « Bence-jones » et protéinurie de 24 heures (H), sur fond de suspicion de « myélome multiple ». En effet, une plasmocytose médullaire à 31% avait été confirmé sur un myélogramme réalisé en amont. L´électrophorèse des protéines sériques créée sur « Cappillarys 2 de Sebia » a mis en évidence un bloc bêta-2-gamma avec une augmentation polyclonale des immunoglobulines (Figure 1). Eu égard au contexte clinique un « Immunotyping sérique » a été pratiqué (Figure 2), ce qui a confirmé la polyclonalité des gammaglobulines. La protéinurie de 24 h était moyenne avec une valeur à 0,680 g/24 h. L´électrophorèse des protéines urinaires, conçue sur « Cappillarys 2 Sebia » a objectivé une discrète protéinurie mixte avec un pic d´allure monoclonale migrant au niveau des gammaglobulines et des traces d´albumine, d´alpha 1 et 2 et de bêta-globulines (Figure 3). L´« Immunotyping urinaire » constitué par la suite n´a montré aucune correspondance avec les immunoglobulines (Ig) : ni les chaînes lourdes (IgG, IgM et IgA), ni les chaînes légères (Kappa et Lambda) (Figure 4). Ce résultat fait donc état d´une absence de Protéines de Bence-jones (PBJ) et d´Ig monoclonales complète. Cependant, un contrôle de l´absence de l'ensemble des Ig a été effectué, spécifiquement celui des IgD et IgE. Pour ce faire une immunofixation urinaire (IF) sur gel d´agarose a été pratiquée sur Hydrasys (Sebia) en utilisant le kit « Hydragel Urine Profil(e) 2/4 Sebia » (Figure 5). Cette dernière électrophorèse a confirmé l´absence réelle de « Protéines de Bence-jones », et d´Ig monoclonale complète dans l´urine reçue. La myoglobinurie était à 2,2 ng/ml avec une myoglobinémie élevée à 91 ng/ml (7-76 ng/ml). À part une bicytopénie avec un taux d´hémoglobine à 10,7 g/dl et une leucopénie, le reste du bilan réalisé était normal : créatinine à 8,6 mg/l soit un débit de filtration glomérulaire calculer selon le MDRD (Modification od Diet in Renal Disease) à 114 ml/min/1,73m^2^; cholestérol total à 3,28 mmol/l; HDL-cholestérol à 1,12 mmol/l; LDL-cholestérol à 1,96 mmol/L; protides totaux à 71 g/l; acide urique à 55 mg/l; et TSHus 1,15 mUI/l. Une seconde électrophorèse des protéines urinaires constituée 1 mois après a permis de constater la disparition du pic d´allure monoclonale au niveau des gammaglobulines (Figure 6.

**Intervention thérapeutique:** la phase analytique au laboratoire a été éffectuée avant la prise en charge thérapeutique du patient. Avant la seconde électrophorèse le patient a bénéficié d´une antibiothérapie ainsi que d´un traitement symptomatique.

**Suivi et résultats des interventions thérapeutiques:** le patient ayant été perdu de vue après sa seconde électrophorèse, n´a pas pu bénéficier d´un traitement adéquat.

## Discussion

**Discussion scientifique:** en diagnostic, le seuil de protéinurie pathologique est définie quantitativement par une excrétion journalière supérieure à 150 mg/24 h (0,150 g/24 h) et/ou par la présence de protéines normalement absente de l´urine [1, 2].

Chez notre patient, la protéinurie était moyenne à 0,680 g/24 h, ce qui a fait suspecter une atteinte rénale minime, compte tenu du contexte clinique de suspicion de « myélome multiple ». L´électrophorèse des protéines urinaire réalisée sur « Cappillarys 2 Sebia » était indispensable pour caractériser les protéines présentes. En effet, elle a objectivé un profil urinaire en faveur de la présence d´Ig monoclonale complète ou celle des chaînes légères (figure 3). Cette caractérisation a permis dans une première interprétation à classer la protéinurie en « protéinurie mixte ». Ce profil électrophorétique était bien inhabituel, car l´immunotyping urinaire conçu afin de déterminer la composition réelle du pic d´allure monoclonale migrant au niveau des gammaglobulines, n´a montré aucune correspondance avec les chaînes lourdes (G, A, M) et légères (Kappa, Lamda) (Figure 4). Par ailleurs, la démarche biologique imposait de vérifier la présence ou l´absence d´IgD ou E qui sont susceptibles d´entrainer de très rares cas de myélomes multiples [3, 4]. Ainsi, l´IF sur gel d´agarose pratiquée sur Hydrasys (Sebia) (figure 5) a objectivé l´absence des IgD et IgE, de PBJ et d´Ig monoclonale complète.

**Discussion de la littérature médicale:** une revue de la littérature nous a permis de suspecter quelques protéines autres que celles habituellement retrouvées dans les urines. En effet, certaines protéines produites en amont du rein entrainent une protéinurie dite « pré-rénale » en passant le filtre du fait de leur faible masse moléculaire dans les urines en l´absence de toute lésion organique ou fonctionnelle du rein. Elles réalisent ainsi une protéinurie de surcharge [1, 5]. La présence de ces protéines de surcharge est souvent corrélée à une pathologie spécifique. Il s´agit des « chaînes lourdes et/ou légères des Ig » retrouvées dans les gammapathies monoclonales, du « lysozyme » retrouvé dans les leucémies aiguës myélocytaires (LAM), de la « bêta-2-microglobuline » retrouvée dans les troubles lymphoprolifératifs, de « l´amylase » retrouvée dans les pancréatites aiguës, des « fragments d´hémoglobine » retrouvés dans l´hémolyse intravasculaire, et de la « myoglobine » retrouvée dans les cas de rhabdomyolyse [5]. Compte tenu du contexte de myélome multiple, seulement la présence probable de myoglobine a été suspectée, avec un cas déjà rapporté par Fernández-Solá *et al*. [6]. Aussi des similitudes de profil électrophorétique ont été retrouvées dans l´étude menée par Rostagno A *et al*. avec la myoglobinurie et la myoglobinémie qui ont été dosées [5]. En effet, les auteurs ont mis en évidence la présence de « myoglobine » dans les urines par un « séquençage des acides aminés N terminaux » suivit par une « spectrométrie de masse » chez un patient souffrant de rhabdomyolyse. Chez notre patient le composant homogène inconnu a migré au niveau des gammaglobulines contrairement à celui de Rostagno A *et al*. [5] qui a migré au niveau des bêta-globulines.

**Justification scientifique:** en effet, une myoglobinurie ne peut exister sans rhabdomyolyse, alors que la rhabdomyolyse ne nécessite pas une myoglobinurie macroscopique. Aussi une augmentation de la myoglobine sérique entraine une augmentation de la créatine kinase (CK) sérique [7]. On peut donc conclure à une rhabdomyolyse aiguë méconnue chez notre patient. La myoglobine qui est une petite protéine d´environ 17 500 Da est rapidement filtrée par le glomérule. La myoglobinurie chez un patient sain est inférieure à la limite de détection de la majeure partie des méthodes qui est < 0,4 mg/l. Alors que 2,2 ng/ml de myoglobinurie ne correspond qu´à des traces de myoglobine. Ces traces peuvent s´expliquer par le moment des prélèvements qui ont probablement été réalisés quelques jours après le processus de rhabdomyolyse et la pharmacocinétique de la myoglobine dont la concentration baisse rapidement grâce à sa petite masse moléculaire et de sa demi-vie d´approximativement 2-3 H [5].

Un mois après avoir retrouvé le patient, un second prélèvement a montré l´absence de protéines dans les urines de 24 h. L´électrophorèse des protéines urinaires constituée sur le même échantillon a permis de notifier la disparition du pic d´allure monoclonale au niveau des gammaglobulines (figure 6). Ces résultats confortent l´ensemble des arguments de myoglobinurie transitoire secondaire à une rhabdomyolyse aigüe méconnue.

## Conclusion

L´électrophorèse des protéines urinaires est une analyse de routine, mais aussi une analyse spécialisée selon le matériel utilisé et l´interprétation du spécialiste. Eu égard aux contextes cliniques spécifiques dans lesquels elle est demandée, toutes anomalies atypiques devraient attirer l´attention du biologiste qui se doit alors d´investiguer grâce à des examens complémentaires précis. L´analyse des pics inconnus comme c´est le cas chez notre patient, renseigne sur des symptomatologies, mais aussi des pathologies sous-jacentes, qui sont importantes pour le clinicien. En effet, Ce cas prouve que la myoglobine urinaire peut donner lieu à des pics d´allure monoclonale migrant au niveau des gammaglobulines.
